# Intra-auditory integration between pitch and loudness in humans: Evidence of super-optimal integration at moderate uncertainty in auditory signals

**DOI:** 10.1038/s41598-018-31792-w

**Published:** 2018-09-12

**Authors:** Kyung Koh, Hyun Joon Kwon, Tim Kiemel, Ross H. Miller, Yang Sun Park, Min Joo Kim, Young Ha Kwon, Yoon Hyuk Kim, Jae Kun Shim

**Affiliations:** 1 Department of Kinesiology, University of Maryland, College Park, MD USA; 2 Department of Mechanical Engineering, Kyung Hee University, Yongin-Si, Gyeonggi-do South Korea; 3 Neuroscience and Cognitive Science Program, University of Maryland, College Park, MD USA; 4 Applied Mathematics & Statistics, and Scientific Computation Program, University of Maryland, College Park, MD USA; 5 Department of Physical Education, Hanyang University, Seoul, South Korea; 6 Present Address: Department of Physical Therapy and Rehabilitation Science, University of Maryland, Baltimore, MD USA

## Abstract

When a person plays a musical instrument, sound is produced and the integrated frequency and intensity produced are perceived aurally. The central nervous system (CNS) receives defective afferent signals from auditory systems and delivers imperfect efferent signals to the motor system due to the noise in both systems. However, it is still little known about auditory-motor interactions for successful performance. Here, we investigated auditory-motor interactions as multi-sensory input and multi-motor output system. Subjects performed a constant force production task using four fingers in three different auditory feedback conditions, where either the frequency (F), intensity (I), or both frequency and intensity (FI) of an auditory tone changed with sum of finger forces. Four levels of uncertainty (high, moderate-high, moderate-low, and low) were conditioned by manipulating the feedback gain of the produced force. We observed performance enhancement under the FI condition compared to either F or I alone at moderate-high uncertainty. Interestingly, the performance enhancement was greater than the prediction of the Bayesian model, suggesting super-optimality. We also observed deteriorated synergistic multi-finger interactions as the level of uncertainty increased, suggesting that the CNS responded to increased uncertainty by changing control strategy of multi-finger actions.

## Introduction

The central nervous system (CNS) receives imperfect afferent signals from the sensory system and delivers defective efferent signals to the motor system, leading to movement variability^1,2^. In the CNS, both afferent and efferent signals of the sensory-motor system are corrupted by noise, leading to uncertainty in overall sensorimotor control. For example, when playing a constant note, a violinist tries to produce desired force on a bow needed to produce the constant sound; however, it is nearly impossible to continuously produce the same action or sound or repeat them over multiple trials^3,4^. The violinist also uses the auditory feedback generated from his or her motor actions in order to estimate how the note is being played, but the auditory system provides noisy information, often leading to imperfect state estimation and consequently an erroneous motor task by the CNS^1^.

In the motor domain, the CNS can coordinate multiple motor effectors involved in a particular motor task for a desired motor outcome resulting from individual effectors’ behaviors while compensating each effector’s errors^5–7^. For instance, if a person is asked to produce a constant pressing force of 10 N using four fingers on one hand, individual finger forces are co-varied so as to reduce the variability of the sum of the four finger forces^4,8^. This phenomenon, also known as motor synergy, has been observed in various types of hand and arm movements such as pressing, grasping, and reaching^9–11^ as well as in whole-body movements^12,13^. In the sensory domain, it has been suggested that the CNS is capable of integrating different sources of sensory information to improve overall perception and improve motor outcomes^4,14,15^ or decisions^16^. This is known as optimal integration or Bayesian integration, and the phenomenon has been observed in the integration of multiple sources not only between different sensory systems (i.e. inter-sensory integration)^17–19^, but also between different physical properties within the same sensory system (i.e. intra-sensory integration)^14,20–24^.

In our previous work, we reported that the CNS could optimally integrate feedback on two physical properties of sound (i.e. frequency and intensity), consistent with the Bayesian model^4^. This intra-auditory integration seems to influence the multi-finger actions in a hierarchical manner. Previous studies have suggested that multi-finger actions are controlled in a hierarchical manner with two levels: individual finger (IF) actions at the lower level and virtual finger (VF) actions at the higher level^4,10,25–27^. VF here is an imagined finger producing the same net mechanical effect as all fingers together. In our previous work, we demonstrated that enhancement of VF control (i.e. motor performance of the VF) could be achieved by improving synergistic IF actions (i.e. motor synergy) through integration of frequency and intensity of sound following the Bayesian model^4^. However, it remains unknown whether the Bayesian model can predict intra-auditory integration for different levels of uncertainty in auditory feedback. In addition to this, it is also unknown how the uncertainty affects multi-finger actions in a hierarchical organization.

Therefore, the current study investigated how the CNS deals with uncertainty manifested by the auditory feedback gains during constant multi-finger force production. Subjects were asked to produce a constant force using four fingers in three different auditory feedback conditions, where either the frequency (F), intensity (I), or frequency and intensity (FI) of an auditory tone changed depending on the deviation of the VF force from a target reference force. We hypothesized that 1) performance would be enhanced in the FI condition compared to the F condition or I condition alone for all uncertainty levels, following the Bayesian model, and 2) synergistic multi-finger action would deteriorate as the level of uncertainty increases, leading to reduced performance consistent with the findings from our previous work^4^.

## Methods

### Participants

Ten healthy right-handed male volunteers (mean age 24.5 years ± 1 year) participated in the study. The sample size was determined by power analysis for statistical analysis conducted in G-Power with an alpha of 0.05, power of 0.95, an effect size of 0.4 and 11 degrees of freedom^28^. Participants were free of neurological disorders, psychiatric disorders, speech-language disorders, hearing impairments, and motor impairments. In order to avoid a potential confounding factor that musical training can cause in terms of auditory-motor integration, participants who had musical training within 5 years were excluded. Participants provided written informed consent. All procedures were approved by the University of Maryland College Park Institutional Review Board. Experiments were carried out in accordance with approved guidelines.

### Experimental setup

Four finger pressing forces were collected using load cells (ATI Nano 17, ATI Industrial Automation, Apex, NC, US) at a sampling frequency of 1,000 Hz with data acquisition hardware (6024E, National Instruments Corporation, Austin, TX, US) using a custom program written with LabVIEW (LabVIEW 8.2, National Instruments Corporation, Austin, TX, US). This program interfaced with a function generator (Agilient 33522 A, Keysight Technologies, Inc., Santa Rosa, CA, US) to register the IF forces and calculate the VF force as the sum of IF forces. The program also generated auditory signals played through left and right ears of headphones worn by the subjects (AE2, Bose Corporation. Framingham, MA, US).

In order to minimize distortion of sound due to headphone frequency response characteristics^29^, the auditory signal was calibrated to produce a constant intensity across all frequencies. Calibration was performed in a soundproof room by manipulating frequency from 20 to 20,000 Hz in 1 Hz increments and normalizing intensity at each increment^4^.

### Task procedures

**Figure 1:**
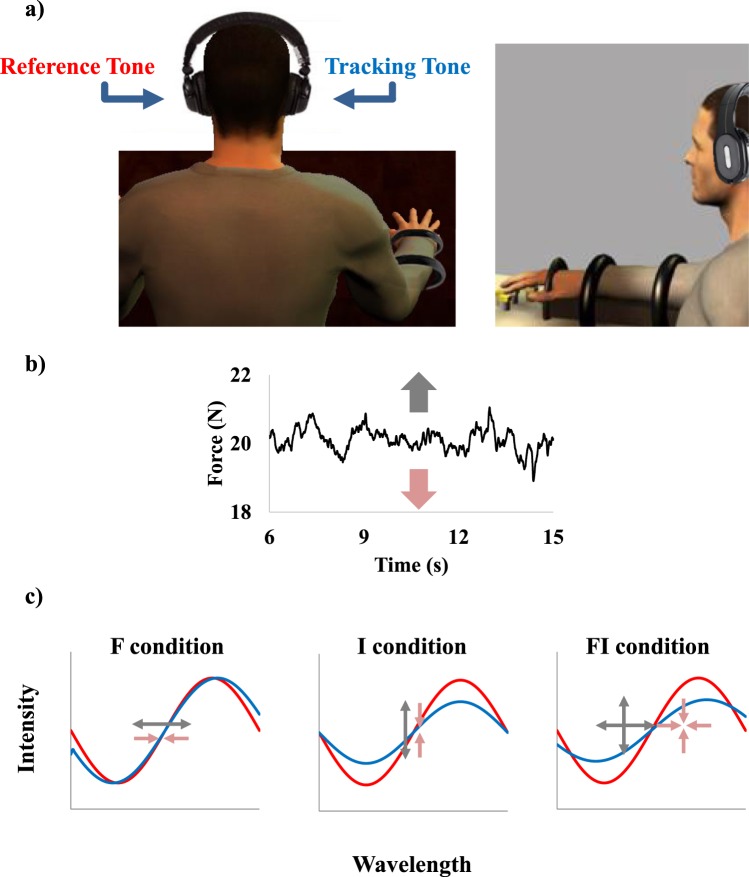
Experimental setup. The subjects sit and place their right-hand finger tips on the sensors while wearing headphones (**a**). The subject is asked to produce 20 N with four fingers while the reference and tracking forces produced are provided as auditory feedback tones. The reference tone (i.e. auditory feedback for reference force) in red (**c**) is a sinusoid signal with a constant frequency of 1000 Hz and intensity of 70 dB played in the left ear, while the tracking tone (i.e. auditory feedback for the tracking force) in blue (**c**) is a sinusoid signal determined by three feedback conditions; Frequency condition (F): the frequency of the tracking tone changed depending on the sum of finger forces, with a constant intensity of 70 dB, 2) Intensity condition (I): the intensity of the tracking tone changed depending on the sum of finger forces (**b**), with a constant frequency of 1000 Hz, and 3) Frequency & Intensity condition (FI): both the frequency and intensity of the tracking tone changed depending on the sum of finger forces.

Subjects sat on a chair, wore the headphones, and placed the tips of their right-hand fingers (index, middle, ring, and little) on the load cells (Fig. 1). The subjects were asked to use these fingers to produce a constant VF force of 20 N (~20% of a typical healthy participant’s maximum voluntary force^4,30^) over 20 s while they received auditory feedback tones of the reference force through the left ear and the VF force through the right ear. The tone for the reference force (i.e. the reference tone) had a frequency of 1000 Hz and intensity of 70 dB^4^. The tone for the VF force (i.e. the tracking tone) played through the right ear varied in three different experimental conditions:

1. Frequency condition (F): the frequency of the tracking tone was modulated with deviation of the subject’s VF force from 20 N, while the intensity of the tracking tone was kept constant at 70 dB^31^.
2. Intensity condition (I): the intensity of the tracking tone was modulated with deviation of the subject’s VF force from 20 N, while the frequency of the tracking tone was kept constant at 1000 Hz^32^.
3. Frequency and Intensity condition (FI): both frequency and intensity of the tracking tone was modulated with the subject’s VF force.

In order to present different levels of uncertainty in the auditory feedback of the VF force, we manipulated the auditory feedback gain for each of feedback conditions (F, I, and FI). For the baseline condition, the gain for frequency and intensity conditions were set as 7 Hz/N and 0.7 dB/N, respectively, according to previous studies on Just Noticeable Differences^4,31,32^. Four gains were used for frequency modulation (300, 86, 24, and 7 Hz/N) and four gains for the intensity (7.5, 3, 1.2, and 0.7 dB/N), which were categorized as low (L), moderate low (ML), moderate high (MH), and high (H) uncertainty conditions. For both frequency and intensity modulations, four gains ([300 Hz/N, 7.5 dB/N], [86 Hz/N, 3 dB/N], [24 Hz/N, 1.2 dB/N], and [7 Hz/N, 0.7 dB/N]) were used. Gains were obtained by the same increments in log scale from baseline conditions to 300 Hz/N for frequency conditions and at 7.5 dB/N for intensity conditions, demonstrated to provide maximum performance. Participants completed 5 trials of 20 s per each condition with 30-s rest between consecutive trials. Prior to the experiment, each participant performed 5 familiarization trials.

From each 20-s trial, the 9-s window from 6 to 15 s, typically capturing the steadiest VF force, was extracted for analysis to avoid the initial force stabilization in the beginning and the premature cessation of force production at the end of each trial^8^. The order of conditions was balanced across subjects.

### Bayesian model

Bayesian model has been a successful in interpreting mechanisms of multi-sensory integration both within a sensory system^4,17–19^ and between different sensory systems^14,20–24^. This model can be useful for investigating the performance enhancement during a particular task where each sensory information provides the same state of physical property because the model can predict performance enhancement. Using the framework of the Bayesian model, the bimodal estimate, ${\hat{S}}_{FI}$, of a finger force from FI can be expressed as a weighted sum of variances from F and I, ${\hat{S}}_{F}$ and ${\hat{S}}_{I}$, respectively;

$${\hat{S}}_{FI}={{\rm{w}}}_{{\rm{F}}}{\hat{S}}_{F}+{{\rm{w}}}_{{\rm{I}}}{\hat{S}}_{I}$$

If the estimates are considered Gaussian random variables with mean $\,\mu$ and variance ${\sigma }^{2}$, the optimal estimate is more precise (lower variance) than the uni-modal estimates as follows:

$${\sigma }_{FI}^{2}=\frac{{\sigma }_{F}^{2}{\sigma }_{I}^{2}}{{\sigma }_{F}^{2}+{\sigma }_{I}^{2}}$$

The variance of combined estimate (${\sigma }_{FI}^{2}$) is lower than the variance from F (${\sigma }_{F}^{2}$) as well as the variance from I (${\sigma }_{I}^{2}$). The combined bias ${b}_{FI}$ (=(${f}_{T}-{\mu }_{FI}$), where ${f}_{T}$ is a reference force (20 N here)), is expressed by a weighted average of the F bias (${b}_{F}$) and the I bias (${b}_{I}$) with the weights ${{\rm{w}}}_{{\rm{F}}}=\frac{{\sigma }_{I}^{2}}{{\sigma }_{F}^{2}+{\sigma }_{I}^{2}}$ and ${{\rm{w}}}_{{\rm{I}}}=\frac{{\sigma }_{F}^{2}}{{\sigma }_{F}^{2}+{\sigma }_{I}^{2}}$^33,34^:

$${b}_{FI}={{\rm{w}}}_{{\rm{F}}}{b}_{F}+{{\rm{w}}}_{{\rm{I}}}{b}_{I}$$

To test whether auditory modalities are optimally integrated according to the Bayesian model, we quantified motor performance in the form of the overall mean-squared error (*OMSE*), the averaged squared deviation of the VF force from the reference force:

$$OMSE=\frac{1}{N}\sum _{i=1}^{N}\{\frac{1}{\tau }{\int }^{}{[{f}_{T}-{y}_{i}(t)]}^{2}dt\}$$

where ${y}_{i}(t)$ is the VF force at trial *i*, and $\tau$ is the duration of ${y}_{i}(t)$.

Then, we compared the experimentally obtained *OMSE* to the *OMSE* predicted by the Bayesian model, which is divided into variable error (${\sigma }_{FI}^{2}$) and systematic error (${b}_{FI}^{2}$) as follows:

$$\begin{array}{rcl}OMS{E}_{FI} & = & {\sigma }_{FI}^{2}+{b}_{FI}^{2}\\  & = & \frac{{\sigma }_{F}^{2}{\sigma }_{I}^{2}}{{\sigma }_{F}^{2}+{\sigma }_{I}^{2}}+{({{\rm{w}}}_{{\rm{F}}}{b}_{F}+{{\rm{w}}}_{{\rm{I}}}{b}_{I})}^{2}\end{array}$$

### Hierarchical variability decomposition model

**Figure 2:**
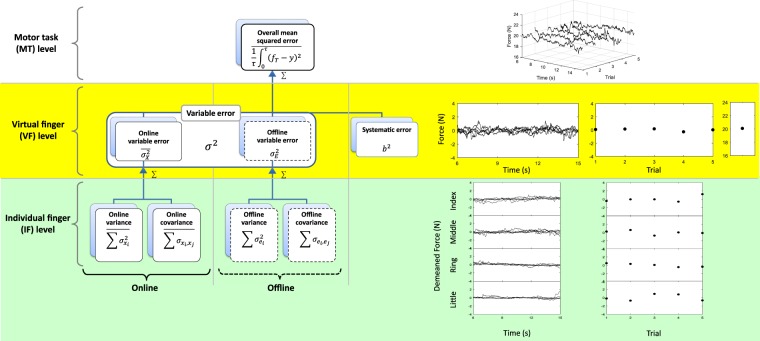
Hierarchical organization of multi-finger force analysis and its corresponding force signals of one representative subject. The overall mean squared error (*OMSE*), shown at the motor task (MT) level, is an averaged squared deviations of VF force from 20 N. At the virtual finger (VF) level, *OMSE* is the linear sum of the variable error ($VE:{\sigma }^{2}$) and systematic error (*SE*: ${({f}_{T}-m)}^{2}={b}^{2}$). The VE (${\sigma }^{2}$) is further decomposed into the intra-trial moment-to-moment (online) variable error (*VE*_*ON*_: $\overline{{\sigma }_{X}^{2}}$) and the time-averaged trial-to-trial (offline) variable error (*VE*_*OFF*_: ${\sigma }_{E}^{2}$). Both the *VE*_*ON*_ and the *VE*_*OFF*_ at the VF level are the linear sums of individual finger (IF) force variances (*Var*_*ON*_: $\overline{{\sum }^{}{\sigma }_{{x}_{i}}^{2}}$ and *Var*_*OFF*_: ${\sum }^{}{\sigma }_{{e}_{i}}^{2}$) and between-finger force covariances (*Cov*_*ON*_: $\overline{{\sum }^{}{\sigma }_{{x}_{i}}{\sigma }_{{x}_{j}}}$ and *Cov*_*OFF*_: ${\sum }^{}{\sigma }_{{e}_{i}}{\sigma }_{{e}_{j}}$) at the individual finger (IF) level. Force signals from a representative subject are presented on the right side of the figure. At MT level, the VF forces are shown again time and trials. At VF level, three components of the VF force, online signal against time, offline signal against trials, and time- and trial-averaged force are shown. At the IF level, these online and offline signals are further decomposed into online and offline individual finger force signals, respectively.

In our previous work, we developed a hierarchical variability decomposition (HVD) model to quantify the hierarchical organization of multi-finger actions in terms of the VF and IF forces (Fig. 2). The VF force for trial *i*, ${y}_{i}(t)$, was modeled as the sum of three components^8^:

$${y}_{i}(t)={X}_{i}(t)+{E}_{i}+m$$

where ${X}_{i}(t)$ is the demeaned VF force for trial *i*, $m$ is the mean VF force after averaging over all timesteps of all 5 trials, and ${E}_{i}$ is the difference between *i*^th^ trial mean VF force and *m*. In this model, *OMSE*, the index of motor performance, was partitioned into three error components as different performance variables^8^:

1. The “online intra-trial variable error (*VE*_*ON*_),” $\overline{{\sigma }_{X}^{2}}$, calculated as the averaged variance of ${X}_{i}(t)$
2. The “offline inter-trial variable error (*VE*_*OFF*_),” ${\sigma }_{E}^{2}$, calculated as the variance of ${E}_{i}$
3. The “systematic error (*SE*) or bias”,$\,{b}^{2}$, calculated as $\,{(20-m)}^{2}$

Note that the sum of online and offline variable errors (*VE*) is the variance of VF force ($\overline{{\sigma }_{X}^{2}}+{\sigma }_{E}^{2}={\sigma }^{2}$), and the systematic error is the squared bias of VF force (${(20-m)}^{2}$ = ${b}_{FI}^{2}$).

The online and offline variable errors can be further defined as the sum of IF variances plus between-finger covariances:

$$\begin{array}{c}\overline{{\sigma }_{X}^{2}}=\overline{\sum _{i=1}^{n}{\sigma }_{{x}_{i}}^{2}}+\overline{\sum _{i\ne j}{\sigma }_{{x}_{i},{x}_{j}}}\\ {\sigma }_{E}^{2}=\sum _{i=1}^{n}{\sigma }_{{e}_{i}}^{2}+\sum _{i\ne j}{\sigma }_{{e}_{i},{e}_{j}}\end{array}$$

where ${x}_{j}$ is the demeaned IF force of the *j*^th^ finger, and ${e}_{j}$ is the IF force difference of the *j*^th^ finger between the means across time and across time steps in 5 trials, n is the number of task fingers (n = 4), and the overhead bars indicate means over trials. The sum of IF variances, *Var*_*ON*_ and *Var*_*OFF*_ ($\,\overline{{\sum }^{}{\sigma }_{{x}_{i}}^{2}}\,$and ${\sum }^{}{\sigma }_{{e}_{i}}^{2}$), reflects the total amount of variability in the motor task, while the sum of IF covariances, *Cov*_*ON*_ and *Cov*_*OFF*_ ($\overline{{\sum }^{}{\sigma }_{{x}_{i},{x}_{j}}}$ and ${\sum }^{}{\sigma }_{{e}_{i},{e}_{j}}$), quantifies synergistic actions between finger forces to attenuate or amplify the VF force error^4,8^.

The indices of synergy quantified above are mathematically equivalent to the index of motor synergy calculated between effectors in the previous studies as the normalized variance difference between task-relevant space and task-irrelevant space initially introduced as the uncontrolled manifold (UCM) analysis^9,13,35^.

### Statistical analysis

All dependent variables were transformed to correct for a non-normal distribution using the log transformation for *OMSE*, *VE*, *SE*, *VE*_*ON*_, *VE*_*OFF*_, *Var*_*ON*_, and *Var*_*OFF*_ and log-modulus transformation methods^36^ for *Cov*_*ON*_ and *Cov*_*OFF*_, which allowed us to transform positive and negative values as follows:

$$T(x)=\,sign(x)\times \,\mathrm{log}(|x|+1)$$

A two-way repeated measures ANOVA with factors *Feedback* (3 levels: F, I, and FI) and *Uncertainty* (4 levels: L, ML, MH, and H) were used to test the differences between conditions. The level of statistical significance was set at *p* = 0.05. A post-hoc test with Bonferroni correction was performed where necessary. We used Greenhouse-Geisser correction for violation of sphericity. Paired t-test with bootstrapping was performed to compare the experimentally obtained *OMSE* to the *OMSE* predicted by the Bayesian model.

## Results

### Comparison with the Bayesian model

**Figure 3:**
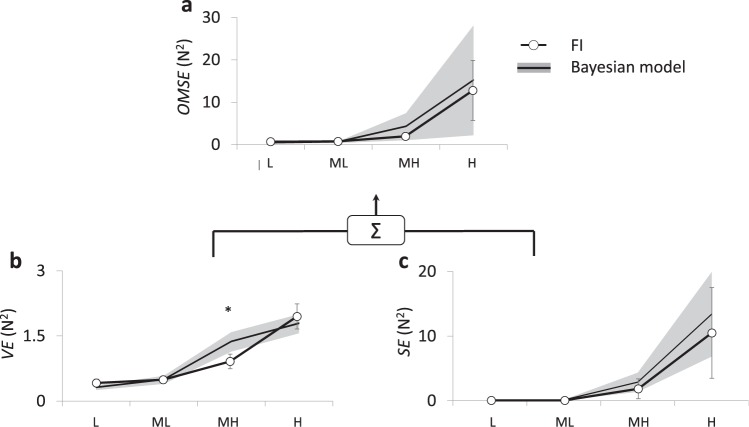
*OMSE*, *VE*, and *SE* from FI (line with circle) are compared with the Bayesian model (line with gray shading) across all feedback uncertainties. The *VE* from FI at MH uncertainty was significantly lower than the Bayesian prediction. The asterisk indicates a significant difference (*≤0.05) among feedback conditions for a given intensity level. Error bars and gray shading represent SEM across subjects.

The Bayesian model well predicts *OMSE* for all different levels of uncertainty. *OMSE* for FI did not differ from *OMSE* estimated from the Bayesian model at any uncertainty conditions (L; p = 0.213, ML; p = 0.625, MH; p = 0.204, and H; p = 0.418) (Fig. 3a), along with no significant differences in *SE* (L; p = 0.484, ML; p = 0.579, MH; p = 0.256, and H; p = 0.405) (Fig. 3c). However, *VE* for FI at MH uncertainty was significantly lower than the *VE* estimated by the Bayesian model (p = 0.037), while there was no significant difference at L (p = 0.212), ML (p = 0.921), or H uncertainty (p=0.424) (Fig. 3b). This result indicates that the CNS improves motor performance exceeding the Bayesian prediction (i.e. super-optimality) when the uncertainty level is moderate.

### Effects of feedback on multi-finger actions in the hierarchical organization

**Figure 4:**
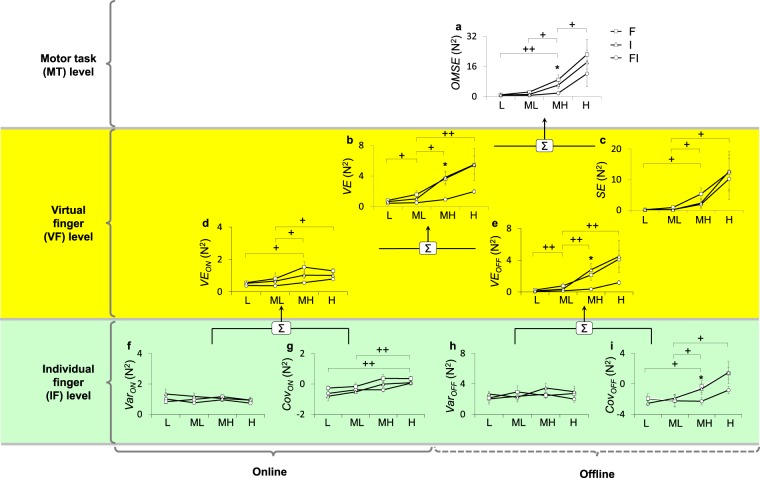
Components of the hiearchical decomposition variability model under F (line with square), I (line with triangle), and FI conditions (line with circle) are shown as a function of uncertainty level. A significant *Feedback* main effect: *OMSE*, *VE*, *VE*_*ON*_, and *VE*_*OFF*_ for FI significantly decreased compared to those for either F or I alone. A significant *Feedback* × *Uncertainty* interaction effect: *OMSE*, *VE*, and *Cov*_*OFF*_ from FI are significantly lower at MH uncertainty compared to either F or I alone. A significant *Uncertainty* main effect: *OMSE*, *VE*, *SE*, *VE*_*ON*_, and *VE*_*OFF*_ (**a**–**e**) significantly increased as uncertainty level increased. At the IF level, *Cov*_*ON*_ and *Cov*_*OFF*_ (**g**,**i**) are significantly increased, while *Var*_*ON*_ and *Var*_*OFF*_ (**f**,**h**) remained unchanged. The asterisk indicates significant difference (*≤0.05) among feedback conditions. The cross indicates significant difference (+≤0.05, ++≤0.01) among uncertainty levels. Error bars represent SEM across subjects.

Multi-finger actions were analyzed at the VF and IF levels using the HVD model. It was found that overall performance quantified as *OMSE* was enhanced through intra-auditory integration. The enhancement was mainly through reduction of the variability in VF force. These results were supported by significant *Feedback* effects on *OMSE* (F_1.193,18_ = 7.832; p = 0.015), *VE* (F_1.197,18_ = 6.809; p = 0.021), and *SE* (F_2,18_ = 3.596; p = 0.049). The pair-wise comparisons showed that both *OMSE* and *VE* from FI were significantly lower than those for both F and I conditions (*OMSE*: FI vs F; *p* = 0.001; FI vs I; *p* = 0.006, and *VE*: FI vs F; *p* = 0.001; FI vs I; *p* = 0.006), while *SE* from FI significantly differed from only that of the F condition (FI vs F; *p* = 0.003, and FI vs I; *p* = 0.607) (Fig. 4b and c). There were significant *Feedback* $\times$ *Uncertainty* interaction effects in *OMSE* (F_6,54_ = 2.412; p = 0.039) and *VE* (F_6,54_ = 2.375; p = 0.041), while no significant *Feedback* $\times$ *Uncertainty* interaction effect was found in *SE* (F_6,54_ = 1.554; p = 0.179). The pair-wise comparisons showed that both *OMSE* and *VE* from FI at MH uncertainty were smaller than those of F or I alone (*OMSE*: FI vs. F; *p* = 0.002; FI vs. I; *p* = 0.006, and *VE*: FI vs. F; *p* = 0.003; FI vs. I; *p* = 0.008).

At the VF level, the performance enhancement for both online and offline controls through intra-auditory integration was observed. This indicates that the CNS combines frequency and intensity of an auditory signal in order to provide more consistent actions within a single trial as well as over multiple trials. This result was supported by a significant *Feedback* effect (*VE*_*ON*_: F_2,18_ = 6.172; p = 0.009, and *VE*_*OFF*_: F_2,18_ = 4.353; p = 0.029). The pair-wise comparisons showed that both *VE*_*ON*_ and *VE*_*OFF*_ from FI significantly were lower than those of either F or I alone (*VE*_*ON*_: FI vs. F; *p* = 0.002, and FI vs. I; *p* = 0.04, and *VE*_*OFF*_: FI vs. F; *p* = 0.009, and FI vs. I; *p* = 0.013) (Fig. 4d and e). There was no significant *Feedback* $\times$ *Uncertainty* interaction effect on either *VE*_*ON*_ (F_6,54_ = 0.866; p = 0.526) or *VE*_*OFF*_ (F_6,54_ = 1.886; p = 0.100).

At the IF level, intra-auditory integration positively affected synergistic actions only in offline control, but not in online control. This indicates that the CNS combines frequency and intensity of an auditory signal in order to provide more consistent actions over multiple trials, not within a single trial. These results were supported by a significant *Feedback* effect on *Cov*_*ON*_ (F_2,18_ = 5.158; p = 0.017), but no significant effect on *Var*_*ON*_ (F_2,18_ = 1.553; p = 0.239), *Var*_*OFF*_ (F_2,18_ = 1.070; p = 0.364), or *Cov*_*OFF*_ (F_2,18_ = 3.333; p = 0.059). There was a significant *Feedback* $\times$ *Uncertainty* interaction effect on *Cov*_*OFF*_ (F_6,54_ = 2.601; p = 0.027) but no significant interaction effect on *Var*_*ON*_ (F_6,54_ = 0.797; p = 0.576), *Var*_*OFF*_ (F_6,54_ = 0.739; p = 0.621), or *Cov*_*ON*_ (F_6,54_ = 0.902; p = 0.501). The pair-wise comparisons showed significant reduction of *Cov*_*OFF*_ from FI compared to that of either F or I alone (FI vs. F; *p* = 0.048, and FI vs. I; *p* = 0.035) in the MH condition.

### Effects of uncertainty on multi-finger actions in the hierarchical organization

As we expected, motor performance quantified as *OMSE* decreased as the uncertainty level increased, which was supported by the significant *Uncertainty* effect (F_3,27_ = 64.074; p < 0.001). The pair-wise comparisons showed significant statistical differences between feedback conditions (L vs, MH: *p* < 0.001, ML vs, MH: *p* = 0.002, MH vs, H: *p* = 0.013) (Fig. 4a). Both *VE* and *SE* increased as uncertainty increased, which was supported by the significant *Uncertainty* effect (*VE*: F_3,27_ = 73.023; p < 0.001, *SE*: F_3,27_ = 23.110; p < 0.001). The pair-wise comparisons showed significant statistical differences between feedback conditions (*VE*: L vs. ML: *p* = 0.016; ML vs. MH: *p* = 0.002; ML vs. H: *p* < 0.001, and *SE*: L vs. MH: *p* = 0.002; ML vs. MH: *p* = 0.010; ML vs. H: *p* = 0.002) (Fig. 4b and c). In the HVD model, *VE* was further partitioned into online variable error (*VE*_*ON*_) and offline variable error (*VE*_*OFF*_) in the VF actions. Both *VE*_*ON*_ and *VE*_*OFF*_ decreased as uncertainty increased, which was supported by the significant *Uncertainty* effect (*VE*_*ON*_: F_1.866,27_ = 23.418; p < 0.001, and *VE*_*OFF*_: F_3,27_ = 85.255; p < 0.001). The pair-wise comparisons showed significant statistical differences between feedback conditions (*VE*_*ON*_: L vs. MH: *p* = 0.004; ML vs. MH: *p* = 0.005; ML vs. H: *p* = 0.005, and *VE*_*OFF*_: L vs. ML: *p* < 0.001; ML vs. MH: *p* = 0.001; ML vs. H: *p* < 0.001) (Fig. 3e).

At the IF level, interestingly, both *Var*_*ON*_ and *Var*_*OFF*_ remained unchanged, while both *Cov*_*ON*_ and *Cov*_*OFF*_ increased from negative values as uncertainty increased. This result indicates that the CNS utilizes multiple fingers in the same workspace, but changes control strategies in response to different uncertainty conditions. The results were supported by the significant *Uncertainty* main effects (*Var*_*ON*_: F_3,27_ = 1.441; p = 0.253, *Var*_*OFF*_: F_3,27_ = 2.646; p = 0.069, *Cov*_*ON*_: F_3,27_ = 17.931; p < 0.001, and *Cov*_*OFF*_: F_1.815,27_ = 20.608; p < 0.001) (Fig. 3f–i) The pair-wise comparisons showed significant statistical differences in both *Cov*_*ON*_ and *Cov*_*OFF*_ between feedback conditions (*Cov*_*ON*_: L vs. H: *p* < 0.001; ML vs. H: *p* < 0.001, and *Cov*_*OFF*_: L vs. MH: *p* = 0.045; ML vs. MH: *p* = 0.001; ML vs. H: *p* = 0.002).

## Discussion

The aim of this study was to investigate the role of auditory feedback uncertainty manifested by auditory feedback gains during a constant multi-finger force production task in three different sound feedback conditions (F, I, and FI). Using the HVD model, multi-finger actions were hierarchically analyzed at the VF and IF levels. First, we expected that intra-auditory integration would occur as evidenced by decreased variable error (*VE*), according to the Bayesian model. However, at MH uncertainty, there was greater reduction in *VE* in the FI condition compared to the Bayesian prediction. Second, we expected that synergistic actions would decrease as uncertainty increased. Indeed, we found that the indexes of synergistic actions (i.e. *Cov*_*ON*_ and *Cov*_*OFF*_) decreased as uncertainty increased, but there were no changes in total variability (i.e. *Var*_*ON*_ and *Var*_*OFF*_).

### The role of auditory uncertainty in intra-auditory integration

The Bayesian model has been used to investigate how the brain integrates multiple sources of sensory information^14,17–24^. These previous studies have suggested that the CNS combines multiple sensory modalities to enhance the state estimate and minimize variability in performance of goal-directed motor tasks to generate “optimal” outcomes. According to the Bayesian model, it might not be possible to produce better outcomes that what is predicted from the model (i.e. “super-optimality). Super-optimal inter-sensory integration has been observed in previous studies on humans and animals^37,38^. Our study also found that the performance improvement through intra-sensory integration was similar to or better than the statistically optimal performance predicted by the Bayesian model. The enhancement of motor performance in our study exceeded the Bayesian prediction when uncertainty was moderately high (Fig. 3b). Although this finding warrants further investigation, one can logically speculate that uncertainty plays a critical role in intra-sensory integration.

The inverse effectiveness rule has also been used to interpret the effects of uncertainty on integration of multiple sensory sources^39–42^. This rule supports the idea that multi-modal feedback is effectively integrated when the uni-modal responses are relatively weak^39^. Greater neuronal responses have been found in multi-modal (visual + auditory) stimulus compared with uni-modal stimulus of a smaller intensity, suggesting that multi-modal integration is inversely related to the intensity of its uni-modal stimulus^40–42^. However, the super-optimality observed in our study deviates from the inverse effectiveness rules because intra-auditory integration was most effective at the intermediate level of uncertainty in our study.

Two physical quantities (frequency and intensity) are the most salient features of sound that contribute to its perception as pitch and loudness. According to the auditory perception theories^43,44^, these two quantities can be independently perceived by the CNS. We perceive and identify the different frequency of sound through the neural response of hair cells in different locations of basilar membrane^45^. On the other hand, the loudness of sound is perceived by changing of firing rate in the auditory nerve (i.e. firing-rate theory)^45^. For example, when the sound is weak (low intensity), only a small region of the basilar membrane moves sufficiently to evoke spikes. For strong sound intensity, on the other hand, the membrane is displaced by a larger amount, causing evoking spikes even in neighboring nerve fibers. In our experimental design, we manipulated a rate of change in frequency and intensity by the finger force to provide different level of uncertainty in the task. Our main finding of super-optimality at the intermediate level of uncertainty implies neural responses in the auditory nerve that is better than a prediction by the Bayesian integration.

### Online vs. offline controls

Online and offline motor behaviors infer distinct control mechanisms of the CNS control mechanisms in redundant motor systems^4,8,46^ since the former is controlled continuously, and the latter is controlled discretely. We noted enhancement of the repeatability (i.e. offline control) of VF actions through intra-sensory integration, but no enhancement of consistency (i.e. online control). In our previous study^4^, we showed that intra-auditory integration had a greater influence on offline control than online control, consistent with other previous studies^14,47^. These previous studies investigated the integration between different senses (e.g. visual and auditory, visual and tactile) and showed subjects enhanced repeatability when using both senses during repetitive tasks such as estimating the position or size of a target. The results of the current study support the theory that the benefits of multi-sensory integration extend to intra-sensory integration as well as to state estimation and repetitive motor performance.

### The role of auditory uncertainty in hierarchically organized multi-finger actions

Previous studies have shown that multi-finger actions are controlled in a hierarchical manner with at least two levels: individual finger actions at the lower level and virtual finger actions at the higher level^10,25–27^. In the current study, we investigated the hierarchical organization of multi-finger actions using the HVD model that quantifies several aspects of motor performance at the VF level such as estimability (i.e. inverse of *SE*), consistency (i.e. inverse of *VE*_*ON*_), and repeatability (i.e. inverse of *VE*_*OFF*_)^4^. In a constant force production task, the estimability reflects the CNS’s ability to estimate the target force and consistency reflects the CNS’s ability to perform the task on a moment-to-moment basis (i.e. online control), while repeatability reflects the ability to repeat the same task goal on trial-to-trial basis (i.e. offline control). At the IF level, the consistency and repeatability at the VF level can be explained by the sum of variability in IF forces and the co-variability (i.e. motor synergy) among the IF forces. Variability (i.e. *Var*_*ON*_ and *Var*_*OFF*_) and co-variability (i.e. *Cov*_*ON*_ and *Cov*_*OFF*_) reflect the CNS’s “work space” and “control strategy” to perform the task, respectively. Note that positive covariance indicates that the IF forces are co-varied to amplify the VF force and increase the performance error, while negative covariance attenuates the VF force and decreases performance error^8^. Thus, increasing and decreasing covariance reflects deterioration and enhancement of multi-finger synergy, respectively, in constant force production tasks by the VF.

As expected, we found that, at the VF level, estimability, consistency, and repeatability decreased (i.e. error variables increased) as auditory uncertainty increased. The result is consistent with the finding of previous studies that have shown that the uncertainty in visual feedback leads to performance errors during a constant finger force control task^46,48^. However, interestingly, at the IF level, the current study found that the total variance in IF forces remained unchanged for all different levels of uncertainty, while covariance between the IF forces increased from negative values as auditory uncertainty increased. According to the principle of non-individualized control^49^, multiple motor effectors (e.g. muscles, joints, or fingers) are not controlled individually, but are rather united as a task-specific organization. Indeed, in support of the principle, our results indicates that the CNS does not reduce the variability of individual finger forces, but rather changes synergistic patterns between finger forces to coordinate the IF actions in order to enhance performance of the VF actions (i.e. task-specific organization, commonly addressed as “synergies” in contemporary literature)^5,6^.

### Limitations

There are some limitations in the current study. First, the feedback gain for the baseline condition was set according to “just noticeable differences” (JND) previously reported assuming a change in force of 1 N^4,31,32^. The use of feedback gains through individual auditory sensitivity might have provided more accurate subject-specific conditions for the study. Second, although the frequency and intensity of sound might be their most evident physical features, these two quantities might not be independent of each other; previous studies on the anatomy of the auditory system suggest that one can influence the other by showing that the psychophysical transformation from frequency-intensity space to pitch-loudness space was not a homeomorphism^50,51^. The accurate quantifications of both the subject-specific gains and the homeomorphism demand new methods achieved through careful experimental design and modeling.
